# Compassionate Use of the ROCK Inhibitor Fasudil in Three Patients With Amyotrophic Lateral Sclerosis

**DOI:** 10.3389/fneur.2020.00173

**Published:** 2020-03-13

**Authors:** Jan C. Koch, Josua Kuttler, Fabian Maass, Teresa Lengenfeld, Eirini Zielke, Mathias Bähr, Paul Lingor

**Affiliations:** ^1^Department of Neurology, University Medical Center Göttingen, Göttingen, Germany; ^2^Department of Neurology, Technical University of Munich, Munich, Germany

**Keywords:** ROCK inhibition, Fasudil, compassionate use, amyotrophic lateral sclerosis, therapy, SOD1, Rho kinase

## Abstract

The Rho kinase (ROCK) inhibitor Fasudil is a promising drug for a disease-modifying therapy of amyotrophic lateral sclerosis (ALS). In preclinical models, Fasudil was shown to increase motor neuron survival, inhibit axonal degeneration, enhance axonal regeneration and modulate microglial function *in vitro* and *in vivo*. It prolonged survival and improved motor function of SOD1-G93A-mice. Recently, a phase IIa clinical trial has been commenced to investigate the safety, tolerability, and efficacy of Fasudil in ALS patients at an early stage of disease (ROCK-ALS trial, NCT03792490, Eudra-CT-Nr.: 2017-003676-31). Although Fasudil has been approved in Japan for many years for the treatment of vasospasms following subarachnoid hemorrhage and is known to have a favorable side effect profile in these patients, there is no data on its use in human patients with ALS or any other neurodegenerative conditions. Here, we report the first three cases of compassionate use of Fasudil in patients with ALS. Between May 2017 and February 2019, one male (66 years old) and two female (62 and 68 years old) subjects with probable or definite ALS according to the El Escorial criteria (one of the females having a pathogenic SOD1 mutation) were administered Fasudil 30 mg intravenously twice daily over 45 min on 20 consecutive working days. Blood pressure, heart rate and routine laboratory tests were constantly controlled. All three subjects tolerated the Fasudil infusions well without any obvious side effects. Interestingly, the slow vital capacity showed a significant increase in one of the patients. Taken together, we report here the first compassionate use of the ROCK inhibitor Fasudil in three ALS patients, which was well-tolerated.

## Introduction

Amyotrophic laterals sclerosis (ALS) is a rapidly progressive disease characterized by the degeneration of upper and lower motor neurons. It leads to paresis of skeletal and bulbar muscles and may finally affect all motoric functions including walking, grasping, and swallowing but also communication and breathing (1). Life expectancy is between 3 and 5 years after symptom onset. Most patients die from respiratory insufficiency or its complications.

ALS is mostly a sporadic disease except for 5–10% of all cases which have a hereditary background, mainly autosomal dominantly inherited mutations of the five most common ALS genes C9ORF72, SOD1, FUS, TARDBP/TDP-43, and TBK1 (2).

The pathogenesis of ALS is not understood in detail yet. Central pathomechanisms that have been implicated are dysregulated RNA metabolism, oxidative stress, impairment of axonal transport and autophagy (2). Intraneuronal aggregates of the RNA-binding protein TDP-43 are the pathological hallmark of >95% of all ALS cases.

Up to now, only two therapies were proven to have mild disease-modifying effects: Riluzole and Edaravone. Riluzole 50 mg orally twice daily has been the standard of pharmacological care worldwide for more than two decades now (3). In the initial clinical trial it led to a survival benefit of 2–3 months especially in patients with bulbar onset (4). Subsequent register studies suggest stronger effects of up to 1 year median survival benefit if the drug is started early (5). In 2017, Edaravone was approved for treatment of ALS in the USA, Japan, South Korea and later also in Canada and Switzerland based on the results of a phase III trial that showed a 30% slowed decline of the ALS functional rating scale (ALSFRS-R) in a selected subgroup of patients treated with Edaravone as compared to placebo (6). Besides, only symptomatic treatments are available and more efficient pharmacological therapies for ALS are urgently needed.

Inhibition of the serine/threonine kinase Rho kinase (ROCK) was shown to counteract neurodegenerative processes and to foster neuronal regeneration in different animal models of neurodegenerative disease (7). On a molecular level, ROCK inactivates the actin depolymerizing factor cofilin via phosphorylation of LIMK. This results in an increased number of actin filaments and a reduced actin turnover, thus counteracting cell growth and axonal regeneration (7). Besides, ROCK targets other cytoskeletal proteins like ezrin, moesin, tau, MAP2, and CRMP2 that are important for axonal integrity and transport. Moreover, ROCK has pro-apoptotic effects through activation of PTEN, inhibition of mTOR and Akt. Inhibition of ROCK by Fasudil has been shown to modulate microglial phenotypes and to promote the expression of M2 instead of M1 markers upon LPS stimulation (8). Thus, inhibition of ROCK counteracts neuronal apoptosis and axonal degeneration and on the other hand fosters axonal regeneration and modulates microglia activation.

The isoquinoline derivative Fasudil was shown to effectively inhibit ROCK and other kinases like PKA, PKG, PKC, and MLCK. Importantly, it significantly prolonged survival, improved motoric function and induced a regenerative response at the neuromuscular junction accompanied by a modulation of microglial activity in the SOD1-G93A mouse model for ALS (9). Similar results were published in parallel by an independent research group (10). Fasudil is licensed in Japan since 1995 for the treatment of vasospasms following subarachnoid hemorrhage. Several thousands of patients have been treated with Fasudil for this indication since. Besides, several clinical trials for other applications, most frequently in cardiovascular disease including pulmonary hypertension and arterial hypertension have been performed (11). Based on this experience, known side effects are mild and include allergic skin reactions, a slight drop in systolic blood pressure and reversible renal impairment without major safety concerns (12).

Given the promising results of Fasudil treatment in preclinical animal models of ALS and its well-known safety profile for other indications it represents an excellent candidate for re-purposing as a disease-modifying therapy in ALS. A phase IIa clinical trial testing the safety and efficacy of Fasudil in ALS patients has thus just started to recruit patients in February 2019 (13).

From 2017 to 2019, before the start of the trial, we applied Fasudil as compassionate use to three ALS patients. These cases are summarized here. To our knowledge this is the first report of the treatment of a neurodegenerative disorder with Fasudil in human patients.

## Case Reports

### Patient A, Male, 66 Years Old

Patient A first noticed gait problems and a general loss of muscle strength in August 2016. He then developed bulbar symptoms in November 2016 comprising dysphagia, dysphonia and dyspnea accompanied by generalized fasciculations, muscle cramps, paresis, and muscle atrophy of hands and feet. Within 6 months he lost 10 kg of his body weight. In January 2017 he was diagnosed with ALS of the axial form. During the next months he developed a respiratory insufficiency, tetraparesis, general muscle atrophy, and a pseudobulbar syndrome.

In 2010, a diabetes mellitus type 2 had been diagnosed. Besides, he had arterial hypertension, hyperlipidemia and a relative cervical spinal canal stenosis (C 4-6) without signs of myelopathy.

His medication included Riluzole 50 mg bid, Mexiletine 150 mg bid, Saxagliptin 2.5 mg bid, Bisoprolol 5 mg qd, Metformin 1 g bid, HCT 25 mg qd, ASS 100 mg qd, Candesartan 24 mg qd, Amlodipine 5 mg qd, and Zopiclone 7.5 mg qd.

The family history did not reveal any neurological disorders in his relatives.

He was a medical doctor and worked as a radiologist in an independent practice.

In the clinical neurological examination he had a bulbar and pseudobulbar syndrome including marked tongue atrophy and fasciculations of the tongue and there was a paresis of both arms with right and distal predominance, and a distal muscle atrophy of both arms. Besides he had enhanced muscle tendon reflexes, especially the biceps and the quadriceps muscle tendon reflexes were pathologically increased. He was able to walk freely for a maximum of 600 m. The other neurological examination including sensibility, coordination and vegetative system was unremarkable. He was 1.68 m tall and weighed 65 kg.

His respiration was severely impaired due to the neuromuscular weakness. At the beginning of the treatment with Fasudil he had a SVC of 800 ml (26% of normal). Therefore, he used non-invasive BiPAP ventilation every night and when needed also during the day (BiPAP-NIV Trilogy 100 device). Additionally, a cough assist device (E70) was used.

The electrophysiological work-up showed normal nerve conduction studies but clear signs of acute denervation in 3 regions (cervical, throracical, lumbar) in the electromyogram (EMG).

The diagnostic probability according to El Escorial criteria was “probable” with signs of upper and lower motor neuron affection in the bulbar and cervical region. ALSFRS-R was 41 on his first visit in our clinic in April 2017 and decreased to 38 when Fasudil treatment was started 1 month later.

Patient A was treated with Fasudil for 20 consecutive working days from 2017/05/02 until 2017/05/29. He received 30 mg Fasudil (Eril® ampoules) dissolved in 100 ml NaCl 0.9% intravenously over 45 min two times daily (at 8:00 a.m. and 3:00 p.m.).

**Figure 1:**
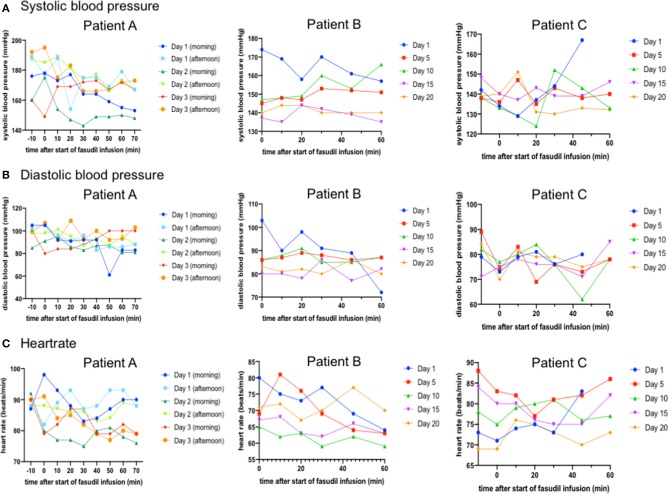
In all patients, arterial systolic **(A)** and diastolic **(B)** blood pressure and heartrate **(C)** were measured at the given time-points before, during and after start of the 45 min Fasudil infusion. In patient A (left column), this monitoring was done regularly on the first 3 days of infusion. Here, all data are shown in the graphs. For patients B (middle column) and C (right column) monitoring was conducted during every infusion. Here, only the data from the morning infusion of every fifth day are given. While patient A and B were hypertonic especially at the beginning of each infusion, no significant or clinically relevant changes in blood pressure or heart rate were noted.

On the first 3 days, blood pressure and heartrate were measured every 10 min before, during and after the infusion of Fasudil (Figure 1). This monitoring was performed since a decrease of arterial blood pressure by a mean of 3 mmHg has been described for Fasudil (Fasudil SMPC). His systolic blood pressure was above normal values at all time points (mean 169 mmHg, range 143–192 mmHg). On average, systolic blood pressure decreased from a mean of 177 mmHg before start of the infusion to a mean of 162.5 mmHg 25 min after the end of the infusion. Diastolic blood pressure (mean 92 mmHg, range 61–107 mmHg) decreased from a mean of 98 mmHg 10 min before the start of the infusion to a mean of 91 mmHg 25 min after the end of the infusion. Heartrate (mean 85/min, range 76–98/min) also showed a slight decrease from a mean of 89/min at 10 min before the infusion to a mean of 83/min at 25 min after the end of the infusion.

As a possible reason the elevated blood pressure, the patient stated he was very excited as he was unsure what to expect from the infusions. Additionally, his previously known arterial hypertension was obviously not sufficiently controlled. With the advice of a cardiologist we therefore increased the dosage of Candesartan to 32 mg qd and recommended further controls and optimization of the antihypertensive medication.

Several times during infusion, standard laboratory parameters were determined from venous blood samples of the patient (Table 1). Hemoglobin levels were slightly below normal but stayed stable during the infusion period (range 12.8–13.4 g/dl). Also, no change was noticed for erythrocyte counts (4.46–4.68 × 10^6^/μl) and thrombocyte counts (196–214 × 10^3^/μl). Leukocyte counts decreased from 7.82 × 10^3^/μl at the beginning to 5.76 × 10^3^/μl at the end of the infusion period but were always within normal ranges. Serum creatinine levels decreased from 1.15 mg/dl at the start to 0.92 mg/dl at the end of the infusions. Glucose levels were above normal (240–297 mg/dl) on the basis of a known diabetes mellitus type 2. Transaminases showed an increase during the infusions (AST 17–23 U/l, ALT 24–31 U/l, GGT 41–54 U/l at the beginning and end of Fasudil infusions, respectively). Surprisingly, creatine kinase increased from 136 U/l at the start to 277 U/l at the end of infusions.

**Table 1:** Laboratory parameters from venous blood samples of the three patients at the given days (V = visit = day of Fasudil infusion) during treatment with Fasudil and at 3 months before and after Fasudil treatment (for patient C).

			**Patient A**				**Patient B**							**Patient C**						
			**Fasudil treatment**				**Fasudil treatment**							**Fasudil treatment**						
	**Range**	**Unit**	**V1**	**V8**	**V15**	**V20**	**V1**	**V6**	**V9**	**V11**	**V13**	**V20**	**+ 3 mon**	**–3 mon**	**V1**	**V3**	**V7**	**V14**	**V20**	**+ 3 mon**
Hemoglobin	11.5–15.0	g/dl	13.1	12.8	13.2	13.4	12.3	13.1	12.2	12.7	12.3	13.0	13.7	14.7	14.5	14.0	14.0	14.4	14.1	14.5
Erythrocytes	3.9–5.1	10^6^/μl	4.68	4.46	4.49	4.57	3.98	4.27	4.09	4.07	4.09	4.25	4.66	4.68	4.65	4.26	4.34	4.33	4.45	4.44
MCV	81–95	fl	86	87	88	89	90	90	91	91	90	90	89	98	99	98	100	99	98	96
MCH	26.0–32.0	pg	28.0	28.6	29.4	29.3	30.8	30.8	29.7	31.1	30.1	30.7	29.4	31.5	31.2	32.8	32.2	33.2	31.6	32.6
MCHC	32.0–36.0	g/dl	32.4	33.0	33.4	33.0	34.4	34.2	32.8	34.3	33.5	34.0	33.2	32.2	31.4	33.3	32.3	33.4	32.2	33.9
Thrombocytes	150–350	10^3^/μl	201	214	196	200	191	210	236	232	245	240	257	285	282	269	272	266	274	285
Leukocytes	4.0–11.0	10^3^/μl	7.82	6.28	6.83	5.76	5.16	5.29	5.48	5.64	5.87	6.03	5.86	6.28	5.59	6.11	6.61	6.21	5.67	6.99
Creatinine	0.50–1.00	mg/dl	1.15	1.06	0.99	0.92	0.49	0.54	0.57	0.39	0.51	0.58	0.48	0.61	0.60	0.55	0.56	0.62	0.60	0.52
Glucose	70–100	mg/dl	240	259	297		91	87	84	108	89	104	92	95	91	85	84	92	81	99
AST	≤31	U/l	17	19	20	23	37	56	37	40	36	36	37	23	24	20	22	22	23	19
ALT	≤34	U/l	24	25	26	31	40	56	42	43	40	41	43	23	22	20	19	21	21	18
GGT	9–36	U/l	41	51	48	54	48	53	46	47	48	51	50	17	18	16	13	14	16	16
Creatin kinase	29–168	U/l	136	220	286	277	165	195	122	121	108	140	144	138	134	148	103	82	71	122

*MCV, mean corpuscular volume; MCH, mean corpuscular hemoglobin; MCHC, mean corpuscular hemoglobin concentration; AST, aspartate transaminase; ALT, alanine transaminase; GGT, gamma glutamyl transpeptidase*.

**Figure 2:**
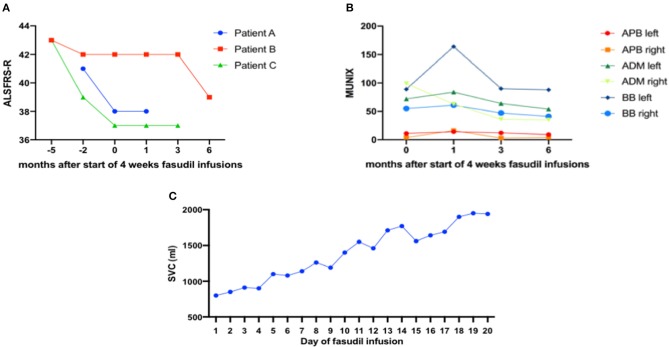
**(A)** Revised ALS Functional Rating Scale (ALSFRS-R) was determined at the given time-points before and after 1 month of Fasudil infusions. Patient A and C had a more aggressive disease progression of >1 point decline per month while patient B showed a decline of only 1 point over 3 months. In all three patients, ALSFRS-R progression was attenuated significantly during and shortly after Fasudil infusions. **(B)** In patient B, Motor unit index (MUNIX) was measured in the given arm muscles before and at the end of 1 month Fasudil infusions as well as 3 and 6 months after the first day of infusion. MUNIX was increased in 5 of 6 measured muscles after 4 weeks of Fasudil treatment. At the follow-up visits at 3 and 6 months, MUNIX gradually decreased again in all measured muscles. APB, abductor pollicis brevis muscle; ADM, adductor digiti minimi muscle; BB, biceps brachii muscle. **(C)** Patient A had a severely impaired respiratory function with a slow vital capacity (SVC) of only 800 ml (26% of normal) at the start of Fasudil treatment. SVC was measured daily during the 20 consecutive working days of Fasudil infusions. It increased to 1,900 ml at the end of the treatment.

During the 4 weeks of Fasudil infusions, no clinical adverse events were noted. The patient stated that he did not notice any adverse effects of the infusions. The infusion procedures were well-tolerated. The neurological examination remained stable, so did the ALSFRS-R which had declined from 41 to 38 points in the month before the infusion but then stayed stable at 38 points until 1 month after the end of Fasudil infusions (Figure 2).

Because SVC was severely impaired, it was measured every day. Interestingly, it increased from 800 ml on the first day of infusion to 1,850 ml on the last day of infusion (Figure 2). At a follow-up examination 4 weeks after the last infusion it remained stable at 1,900 ml.

The motor unit index (MUNIX) was shown to be a very sensitive marker for disease progression in ALS patients (14). Unfortunately, we could only measure MUNIX for patient A once before the Fasudil infusions so no conclusions can be drawn on disease progression.

Unfortunately, patient A was lost to further follow-up as he lived 350 km away from our clinic and was not willing to come for further examinations after the end of infusions. However, we were notified that in the following year his condition worsened severely, especially the respiratory situation. He died in summer 2018, about 14 months after the Fasudil treatment.

### Patient B, Female, 63 Years Old

In patient B, symptoms started in August 2016 with a weakness of the elevation of the left foot leading to gait abnormalities. Symptoms were first attributed to a herniated lumbar disc (L5/S1) which had been operated before in 2006. Over the following months, however, the gait abnormalities worsened and also the elevation of the right foot became weak. A spasticity of both legs could be noticed. In September 2017 the diagnosis of ALS was made.

Interestingly, patient B has a positive family history for ALS: her aunt and her cousin of her father's side were diagnosed with ALS. Her father had died with 37 years due to “pneumonia,” her grandfather died in war. Genetic testing was performed and revealed a heterozygous pathogenic SOD1:c.346C>G [p. (Arg116Gly)] mutation also known as p. (Arg115Gly). Besides, she had two normal C9ORF72 alleles with 2/5 GGGGCC-hexanucleotide repeats.

On clinical examination, patient B had a distally pronounced paraparesis of both legs with marked spasticity and muscle atrophy. The arms were much less affected but also showed muscle atrophy, mild weakness and increased muscle tone and tendon reflexes. There were no bulbar symptoms. Respiration was not affected, the SVC was 4,000 ml (104% of normal). She was 162 cm tall and weighed 68 kg.

Electrophysiological examination showed a pure axonal motor polyneuropathy of arms and legs without evidence of conduction blocks. Besides, a sensory axonal neuropathy of the left sural nerve was noticed. In EMG studies in September 2017, spontaneous firing activity was found in muscles of all 4 regions along with signs of chronic denervation.

MRI-scans of the head and the cervical spinal cord showed no relevant abnormalities. Lumbar puncture yielded normal results.

According to El Escorial criteria the diagnosis of ALS was probable (clinical signs of upper and lower motoneuron pathology in cervical and lumbar regions). Disease progression was relatively slow as ALSFRS-R had been 44 in October 2017 and 42 in August 2018.

Concomitant diseases were hypothyroidism, cervical disk protrusion (C5/6), spondylosis deformans of the thoracic spines and previous herniated disk surgery L5/S1 in 2006.

At the time of Fasudil treatment she was taking the following medication: Riluzole 50 mg bid, Baclofen 10 mg tid, L-Thyroxin 50 μg qd, Magnesium 100 mg bid and vitamins.

Patient B was treated with Fasudil for 20 consecutive working days from 2018/08/20 until 2018/09/14. She received 30 mg Fasudil (Eril® ampoules) dissolved in 100 ml NaCl 0.9% intravenously over 45 min two times daily (at 8:00 a.m. and 3:00 p.m.).

Blood pressure and heartrate were measured every 10 min before, during and after the infusion of Fasudil on every day of the treatment (Figure 1). Mean systolic blood pressure was mildly elevated (mean 147 mmHg, range 126–179 mmHg). There was no significant decrease in blood pressure noted in this patient. Contrary, mean systolic blood pressure increased from a mean of 147 mmHg before start of the infusion to a mean of 151 mmHg at the end of the infusion during the morning infusions. No significant changes in mean systolic blood pressure were noted during the afternoon infusions. Mean diastolic blood pressure (mean 84 mmHg, range 68–113 mmHg) stayed stable during all infusions. Heartrate (mean 70/min, range 59–88/min) also did not show significant changes during and after infusion, but was slightly higher in the afternoon (73/min) than in the morning (67/min).

Laboratory parameters were assessed several times during infusion from venous blood probes of the patient (Table 1). Hemoglobin and blood cell counts were all within normal ranges at all time-points and did not show any significant alterations (erythrocytes 3.98–4.27 × 10^6^/μl, leukocytes 5.16–5.87 × 10^3^/μl). Thrombocyte counts showed a mild increase but within normal ranges (191 × 10^3^/μl at the start of infusions, 240 × 10^3^/μl at the end). Serum creatinine (0.39–0.58 mg/dl) and glucose levels (84–108 mg/dl) were within normal ranges and did not change significantly. Transaminases were mildly increased already before the infusions, probably related to Riluzole, but stayed stable during the Fasudil infusions (range of AST 36–56 U/l, ALT 40–56 U/l, GGT 46–51 U/l). Contrary to patient A, creatine kinase decreased during treatment from 165 U/l at the start to 140 U/l at the end of infusions with even lower levels in the meantime (e.g., 108 U/l at day 13 of infusions).

During the 4 weeks of Fasudil infusions, no clinical adverse events were noted. The infusion procedures were well-tolerated. The neurological examination remained stable. ALSFRS-R stayed stable at 42 points at the start and end of the infusions and at 3 months after the treatment (Figure 2). In a follow-up visit at 6 months after the start of Fasudil treatment ALSFRS-R had declined to 39 points.

ECAS was tested repeatedly and showed a significant increase over time, likely due to learning effects (at start of Fasudil treatment: 121 points of a maximum 136 points, after 4 weeks of Fasudil treatment: 130 points, 3 months later 131 points, and 6 months later 134).

MUNIX was performed at different time points (Figure 2). Interestingly, MUNIX values had increased in 5 of 6 examined muscles at the end of the 4-week infusion period as compared to the first day of treatment indicating an improvement of muscular innervation during Fasudil treatment. At the follow-up visits at 3 and 6 months after Fasudil treatment MUNIX values of all examined muscles gradually showed a mild decrease.

Patient B was last seen at 6 months after Fasudil treatment and at this time-point her fine motor skills of the right hand had mildly worsened and she first noticed some difficulties swallowing. Other than that, symptoms were stable. No long-term adverse effects of the Fasudil treatment had been noted.

### Patient C, Female, 68 Years Old

In June 2016, patient C developed a weakness of the left leg that spread to the left arm within a few months accompanied by muscle atrophies. Fasciculations were noted ubiquitously. Later, a less pronounced weakness and muscle atrophy appeared also on the right side. There were no bulbar symptoms, no dyspnea, no cognitive deficits and no weight loss. In August 2018, the diagnosis of ALS was made and a treatment with Riluzole was initiated. Due to gastro-intestinal side-effects, she did not tolerate the full dose, but only took 50 mg qd which was well-tolerated. Besides, she took Baclofen 5 mg qd and L-Thyroxin 50 μg qd.

Other known conditions were hypothyroidism, lumbar disc herniation and fracture of the right humerus in 2018.

On clinical examination, patient C had a proximally and left-side pronounced tetraparesis with muscle atrophy and fasciculations. The muscle tone of the left leg was increased and muscle tendon reflexes of both arms and legs were pathologically enhanced. The gait appeared spastic-atactic. Free walking distance was 400 m. There were mild bulbar and pseudobulbar symptoms in form of dysarthria, decreased tongue motility, tongue atrophy, and fasciculation of the tongue. Mild dysphagia was noticed accompanied by hypersalivation. ECAS at the beginning of Fasudil infusions was 129 of 136 points. SVC was 2,800 ml (93% of normal). Patient C was 170 cm tall and weighed 75 kg.

Electrophysiological work-up revealed a mild axonal motor polyneuropathy of the legs. In the EMG, pathological spontaneous firing activity was found in the tibialis anterior and vastus lateralis muscles of the left leg, chronic signs of denervation were found in arm, leg, and thoracic muscles. In the motor evoked potentials, the central motor latencies were significantly prolonged to both arms and legs, additionally the peripheral motor latency to the right leg was prolonged. Myosonography showed general muscle atrophy and fasciculations in the tongue, biceps brachii muscle, and several leg muscles. MRI of brain and spinal cord did not show any specific abnormalities. CSF-studies were normal except for a significantly increased neurofilament [pNfH in CSF: 3,996 pg/ml (cut-off for MND: 560 pg/ml) (15)].

The diagnostic probability according to El Escorial criteria was “definite” with clinical signs of upper and lower motor neuron affection in the bulbar, cervical, and lumbar regions. ALSFRS-R was 43 on her first visit in our clinic in August 2018 and decreased to 37 when Fasudil treatment was started 5 months later.

Patient C was treated with Fasudil (Eril®) for 20 consecutive working days from 2019/01/14 until 2019/02/08. She received 30 mg Fasudil dissolved in 100 ml NaCl 0.9% intravenously over 45 min two times daily (at 8:00 a.m. and 3:00 p.m.).

Blood pressure and heartrate were measured every 10–15 min before, during and after the infusion of Fasudil on every day of infusion (Figure 1). Mean systolic and diastolic blood pressure were within normal ranges. Mean systolic blood pressure (mean 139 mmHg, range 112–167 mmHg) decreased after lying down from 141 mmHg 10 min before the start of infusion to 134 mmHg at the start of Fasudil infusions and then stayed stable at around 134 mmHg at all time points during Fasudil infusion. No significant decrease could be noted. Mean diastolic blood pressure (77 mmHg, range 49–102 mmHg) stayed completely stable during all infusions. Heartrate (mean 75/min, range 65–88/min) also did not show significant changes during and after infusion.

Laboratory parameters were assessed several times during infusion from venous blood probes of the patient (Table 1). Hemoglobin and blood cell counts were all within normal ranges at all time-points and did not show any significant alterations (erythrocytes 4.26–4.68 × 10^6^/μl, leukocytes 5.67–6.61 × 10^3^/μl, thrombocytes 266–285 × 10^3^/μl). Mean corpuscular volume (MCV) was increased at all time points (98–100 fl), the reason for this finding is still not clear as folate acid (9.0 μg/l) vitamin B12 (336 ng/l) and holotranscobalamine (129.4 pmol/l) were all within normal ranges and no hematologic disease was known. Serum creatinine (0.55–0.62 mg/dl), glucose (81–95 mg/dl), and transaminases (AST 20–24 U/l, ALT 19–23 U/l, GGT 13–18 U/l) were within normal ranges and did not change significantly. Similarly to patient B, creatine kinase decreased significantly during treatment from 134 U/l at the start to 71 U/l at the end of infusions.

During the 4 weeks of Fasudil infusions, no clinical adverse events were noted. The patient stated that she did not notice any negative effects of the infusions. The infusion procedures were well-tolerated. The neurological examination remained stable, so did the ALSFRS-R at 37 points (Figure 2) and the SVC at 2,800 ml.

MUNIX was performed at the first day of Fasudil infusion and at 3 months follow-up. There was a decrease in some muscles especially on the left hand and both legs (left APB 01/19: 28, 04/19: 5; left ADM 01/19: 33, 04/19: 18; left TA 01/19: 75, 04/19: 66; right TA 01/19: 78, 04/19: 39) while some muscles remained quite stable (right APB 01/19: 94, 04/19: 86; right ADM 01/19: 151, 04/19: 134, left BB 01/19: 40, 04/19: 53; right BB 01/19: 86, 04/19: 88). Unfortunately, no MUNIX was performed at the last day of infusion, when an increase of most MUNIX values had been noted for patient B.

At follow-up at 3 months after the Fasudil infusions, the patient reported no side-effects. ALSFRS-R remained stable at 37 points, however, a mild progression of the muscle weakness on the left side was noticed and free walking distance was further reduced. SVC decreased to 2,610 ml (before 2,800 ml). Laboratory parameters were unremarkable (Table 1).

## Discussion

We report here the first compassionate use of the ROCK inhibitor Fasudil in patients with ALS. Fasudil was applied intravenously 30 mg twice daily on 20 consecutive working days. This corresponds to the maximum dose that is licensed in Japan for the treatment of subarachnoidal hemorrhage. The drug and the infusion procedures were well-tolerated by all three ALS patients. No side-effects were noted. In particular, no significant changes of blood pressure, heartrate, and any laboratory parameters could be found.

No conclusions with regards to efficacy of the treatment can be drawn from these single patients. Symptoms had progressed in all patients at 3 or 6 months follow-up. It is impossible to say whether progression was attenuated compared to the natural course of the disease. There were, however, several signs of a possible transient positive effect of Fasudil. In patient A, SVC increased from 800 to 1,850 ml during the infusion therapy and remained stable at this level for several weeks. Part of this increase might be attributed to a training effect since measurements were repeated daily. However, as the effect showed a constant increase and ended up with more than 100% of the initial SVC, we do not think that training can explain all of this observation. In the other two patients, respiration was not impaired, repeated measurements were not performed and we could not detect any therapeutic effects here. In patient B, MUNIX was performed at the beginning and at the end of Fasudil treatment and showed a consistent increase in almost all muscles indicating a possible regeneration of motor units. However, this effect was only transient, as values decreased again at 3 and 6 months follow-up. In all three patients, the decline of the ALSFRS-R was attenuated during and at 1 month after Fasudil infusions, suggesting a possible positive effect of Fasudil on motor function.

The transient nature of the putative therapeutic effects suggests that Fasudil treatment should be tested over a longer period. Since safety data are only available for a maximum of 12 weeks therapy, this would require previous safety tests in animals. Another disadvantage is the intravenous route of administration, although well-tolerated over the 4 weeks period here. Therefore, future efforts should be made to develop an oral form of Fasudil that can be applied over longer periods.

A multi-centric randomized placebo-controlled phase IIa clinical trial aiming to enroll 102 ALS patients has started in February 2019 (ROCK-ALS trial; EudraCT-Nr. 2017-003676-31) (13). Study drug infusions will be applied similarly to the procedures in the three patients described here. The ROCK-ALS trial will evaluate safety and tolerability as primary endpoints in a larger patient collective. Besides, several secondary endpoints will assess efficacy including ALSFRS-R, MUNIX, SVC, and laboratory parameters including neurofilaments and urinary p75ECD.

In summary, the three ALS patients described here tolerated Fasudil 30 mg i.v. twice daily for 20 days well without any adverse effects. The ongoing placebo-controlled ROCK-ALS trial will further evaluate safety and tolerability as well as efficacy in a larger patient group.

## Ethics Statement

Ethical review and approval was not required for this compassionate use in human participants in accordance with the local legislation and institutional requirements. The patients provided their written informed consent to receive the compassionate use treatment and for publication of the data.

## Author Contributions

JCK and PL planned the treatment and supervised the clinical procedures. JCK wrote the manuscript and analyzed the data. JCK, JK, FM, TL, and EZ performed all clinical and diagnostic procedures. All authors contributed to manuscript revision, read, and approved the submitted version.

### Conflict of Interest

The authors declare that the research was conducted in the absence of any commercial or financial relationships that could be construed as a potential conflict of interest.
